# Advice to a Dyspeptic

**Published:** 1875-04

**Authors:** 


GOOD ADVICE TO A DYSPEPTIC.—A gentleman saw an advertisement that a receipt for the cure of dyspepsia might be had by sending two postage stamps to the advertiser, and the answer was, "Dig in your garden and let whisky alone."

